# The Rising Challenge: Addressing the Pink Eye (Acute Conjunctivitis) Outbreak in Pakistan

**DOI:** 10.34172/aim.28619

**Published:** 2024-07-01

**Authors:** Asif Mahmood, Shama Shama, Wen Zhang

**Affiliations:** ^1^Department of Microbiology, School of Medicine, Jiangsu University, Zhenjiang, China; ^2^School of Material Science and Engineering, Jiangsu University, Zhenjiang, China

 Acute conjunctivitis, commonly referred to as ‘pink eye,’ is one of the most common inflammations of the conjunctiva, a thin, transparent tissue that lines the inner surface of the eyelid and eyeball. It has been an emerging problem in the major cities of Pakistan, beginning in Karachi, and then involving Lahore, Rawalpindi, Islamabad, and other cities.^1^ This ophthalmic infection is a prevalent medical condition not only in Pakistan but also recently in other countries like Vietnam and India. It can affect individuals of all ages and backgrounds and is frequently seen in hospital outpatient departments (OPD).^2^ The eye conjunctiva is usually transparent; it turns pink or red when it gets infected or inflamed. It is also referred to as conjunctivitis, and is characterized by various levels of severity, from slight redness or irritation in the initial days to increased watery discharge from the eyes, and it may persist for 7–15 days with serious symptoms like purulent discharge (pus), subconjunctival hemorrhage, and swollen conjunctiva. Various organisms like bacteria, allergens, and parasites are responsible for conjunctivitis, but about 20%–70% of acute conjunctivitis infection cases are caused by various viruses, and one of them is the human adenoviruses (HAdVs), which account for 65%–90% of severe conjunctival infections known as ‘pink eye’. HAdV-related conjunctival infections can lead to reduced vision, prolonged discomfort, and occasionally severe complications.^3^ HAdVs belong to the genus* Mast-adenovirus* in the family* Adenoviridae* and are categorized into seven distinct species (A-G), with more than 51 serotypes each distinguished by their distinct antigenic characteristics (like biological, immunological, and biochemical), while types 52–68 serotypes are classified using genomic criteria. Among these species, species B includes HAdV-3 and -7, species D encompasses HAdV-8, -19, and -37 species, species E includes HAdV-4, and recently identified genotypes HAdV-53, -54, -56, and -64 of species D are also associated with eye infections.^4^

 In September 2023, the Chief Minister of Punjab, Pakistan, alerted health professionals and the public about a pink eye (acute conjunctivitis) outbreak. More than 86 133 cases of pink eye have been reported since the onset of the autumn season, coinciding with a noticeable drop in temperature.^5,6^ In the current year, a report from the Primary and Secondary Health Department of Punjab revealed 379 690 reported cases of eye infections across 36 districts in Punjab. The city’s air quality worsened due to reduced rainfall this year. Typically, the city witnesses two outbreaks of conjunctivitis during changing weather conditions.^7^ Notably, the total number of cases had doubled since the previous month of August, and its highly contagious disease appears to have impacted young children and middle-aged individuals more, possibly due to their frequent social interactions in educational institutions or other public places where hand hygiene is often neglected. The epidemic outbreak of pink eye (viral conjunctivitis) was first observed in Karachi, then in Lahore, and rapidly spread like wildfire to other cities in the Punjab and KPK provinces in Pakistan.^5,6^ Schools or other educational institutions in the affected regions had to be temporarily closed due to the disease’s rapid transmission caused by frequent contact between students at institutions. This incident constituted one of the most significant pink eye outbreaks in the nation’s recent history.^6^ Common viruses like HAdVs (serotypes 3, 4, 7, 8, 9, 11, 19a, 21, 35, and 37) and enterovirus 70, along with a variant of the Coxsackie virus known as Cox.A24v, are often the cause of acute viral conjunctivitis.^4^ Acute conjunctivitis can appear similar to other conditions, such as conjunctivitis related to allergies, chlamydia infection, acute congestive glaucoma, and the recent COVID-19 infection. Hence, using different terms for eye conditions like “pink eye,” “red eye,” or “eye flu” in the media can create confusion among people.^1^

 Lack of evidence should not result in ignoring a problem. Even though acute viral conjunctivitis tends to resolve on its own and there might not be a lot of clinical proof, we should not underestimate its impact. This is especially important given the lack of updates on the viral conjunctivitis outbreak after it occurred.^1^ Viral conjunctivitis makes up 70%–80% of acute conjunctivitis cases. If viral conjunctivitis is mistakenly diagnosed as bacterial conjunctivitis and antibiotics are used unnecessarily, it could worsen the problem of antibiotic resistance.^8^ In Pakistan, many infected individuals do not consult doctors and tend to self-medicate, which can harm their eyes. The disease is highly contagious, similar to COVID-19, spreading primarily through direct contact with an infected person rather than solely through eye contact. Lack of awareness and excessive screen time on mobile phones can worsen the condition. Furthermore, incorrect treatment could aggravate conditions like epidemic keratoconjunctivitis, acute hemorrhagic conjunctivitis, and pharyngoconjunctival fever, potentially harming eyesight (Table 1).

**Table 1:** Salient Aspects of Different Types of Pink Eye Conjunctivitis, Their Causes and Treatment

**Pink Eye Conjunctivitis**			
**Types of Pink Eye Conjunctivitis**	**Sign and Symptoms**	**Causal Viruses**	**Treatment**
Epidemic Keratoconjunctivitis (EKC)	Redness or irritation Watery discharge (Epiphora) Foreign body sensation Blurred vision Photophobia Swelling or edema of the conjunctiva Preauricular lymphadenopathy Subepithelial infiltrates	Human adenoviruses (HAdVs) species D causes EKC (serotypes 8, 19, 37,53, 54, 56, 64, and 85)	Antibiotic drops Steroids drops
Acute-hemorrhagic conjunctivitis (AHC)	Sudden onset Pain and discomfort Redness Itching and irritation petechiae follicular conjunctivitis, lid edema photophobia Blurred vision	Enterovirus 70 (EV70) Coxsackie A24	Antibiotic eye drops Steroids eyedrops Immune suppression eyedrops
Pharyngo-conjunctival fever (PCF)	Fever Sore-throat Epiphora Red eyes Chemosis (swelling edema) Follicular conjunctivitis, Swollen lymph nodes General Malaise Nasal congestion	Human adenovirus serotype2,3,4,7 and 14. Coxsackie virus A24 variant (Cox.A24v)	Artificial tears Antibiotic drops
Acute non-specific follicular conjunctivitis (ANFC)	Redness Irritation Lacrimation Soreness (mild pain) Tarsal follicles Swelling Photophobia	Adenovirus serotypes 8, 19, and 37	Antiviral drops Antibiotic drops
Chronic papillary keratoconjunctivitis (CPC)	Redness Lacrimation, Photophobia Itching Blurred vision. Conjunctival papillae	Adenovirus types 3, 4, 7, and 14	Artificial tears Anti-allergic drops Mild steroids eyedrops

 The provincial government should cooperate with major eye care hospitals to offer teleophthalmology services via mobile telemedicine vans and apps in rural areas, limiting patients’ movement to prevent the spread of infection. Additionally, healthcare professionals like pharmacists and nurses, particularly in resource-limited areas, can facilitate communication between patients and healthcare providers. Conducting extensive PCR-based DNA sequencing can enhance surveillance efforts. In response to rising cases, the Primary and Secondary Healthcare Department in Punjab issued a public advisory emphasizing safety measure such as using hand sanitizers, avoiding touching the eyes without proper hand hygiene, and refraining from using items that have come into contact with infected individuals.

 Furthermore, it is crucial to recognize that when the government and health establishments are not proactive and there is lack of public awareness during a preventable conjunctivitis outbreak, it not only affects patients and their caregivers but also places a significant social and economic burden. Moreover, it hampers the goal of achieving universal health coverage.
